# Supplementary material for: The Potential microRNA Prognostic Signature in HNSCCs: A Systematic Review
Source: Noncoding RNA. 2023 Sep 14;9(5):54. doi: 10.3390/ncrna9050054 (PMC10514860; doi:10.3390/ncrna9050054)
Supplement: Supplementary file 1 [file ncrna-09-00054-s001.zip › ncrna-2517628-supplementary.pdf]

"cutoff value" "p value"
82073.6666666667 0.496654205496861
82822.3333333333 0.445460420584792
82874.3333333333 0.378114067718528
82952.6666666667 0.45405933041629
83220.3333333333 0.425266760027564
83281.3333333333 0.360350231042263
83360 0.417368740121821
83368.6666666667 0.353602045039084
83495.6666666667 0.324340749736866
83712.6666666667 0.294633296954918
84137.6666666667 0.253130080753877
84139 0.324714307959599
84360.3333333333 0.355150373732316
84456.6666666667 0.33110449034668
84544.3333333333 0.287698805743932
84601 0.336394119524748
85103.6666666667 0.31532979493815
85245.6666666667 0.314769124038461
85257 0.282019242697475
85420.6666666667 0.344622267801741
85475.6666666667 0.423476900479705
85746.6666666667 0.38321285017273
85855.6666666667 0.344706798883582
86063.6666666667 0.326000121171092
86487.3333333333 0.403011721689044
86558 0.384979075297523
86577 0.334371355553705
86782.6666666667 0.333939270755168
86862.3333333333 0.287222772342023
86940.3333333333 0.250354861625512
87077 0.226166214576547
87254.6666666667 0.203763286719611
87284.6666666667 0.200750444291658
87684 0.173697221136127
87756.3333333333 0.166807326535295
87765.6666666667 0.198547790199506
87946.3333333333 0.169566918844272
88095 0.216606400196792
88630.6666666667 0.178520062845605
88993.3333333333 0.222637827019641
89051.6666666667 0.20251335395389
89069 0.219430615376442
89216.3333333333 0.214450197534526
89297.6666666667 0.192782106012711
89401.6666666667 0.241577132792289
89458.3333333333 0.219694460058572
89495 0.247431089926002
89563.6666666667 0.308239043888176
89631 0.277728284087636
89793.3333333333 0.232353261399607
89810.3333333333 0.192670466898962
89828.3333333333 0.170960978606887
89953 0.204763265716614
90002 0.260930405824504
90064.3333333333 0.260103583832532
90264.3333333333 0.220667015582418
90365 0.200811443709181
90505.3333333333 0.245586675407976
90648 0.283869736007025
91378 0.238177456866502
91457.3333333333 0.216223134863292
91701.3333333333 0.185076883602379

91977.3333333333 0.157004832054077
92299 0.141658892069749
92414 0.152617487474037
92753.3333333333 0.13393817801869
93467.6666666667 0.10849418606676
93986.3333333333 0.132384729331164
94070.3333333333 0.11782474886568
94246 0.09975796704861
94267 0.128746428150628
94571.6666666667 0.115579664307538
94812.6666666667 0.14085261244206
94947.6666666667 0.137957261485906
95082 0.114601330668906
95351 0.150194212555171
95361.6666666667 0.193838995498738
95529.3333333333 0.228635779327385
95531.6666666667 0.208179945931881
95533.6666666667 0.172368872925764
95621.3333333333 0.141433466387337
95776 0.170333721575741
95915.6666666667 0.157559780757176
95983 0.200005344349327
96037 0.171918407129354
96199.3333333333 0.209793808615438
96284.6666666667 0.189736724113236
96361.3333333333 0.241895504736853
96719.6666666667 0.221254633983208
96935.3333333333 0.277548762215259
97227 0.246552679096391
97245 0.206041387030199
97442.6666666667 0.243562054576845
97558 0.226423182849142
97600.6666666667 0.282944323130399
97682.3333333333 0.238201269351344
97741 0.208055923224599
98147 0.237344500224883
98226 0.198012941557556
98283 0.24513263342983
98323.6666666667 0.212051362469077
98405.3333333333 0.248464801453379
98874 0.285606436526541
98894 0.312858454816094
98907 0.282147156559399
99189 0.24434935982904
99289 0.243026210236394
99510.3333333333 0.208504985090427
99938 0.256161086320321
100059 0.304682118013417
100156 0.358514214667188
100239.333333333 0.412714312528936
100775.333333333 0.497151907931204
100813.666666667 0.585744816006324
100995.333333333 0.560925481654699
101025.333333333 0.519331069830723
101041 0.472722781305634
101189.333333333 0.432813074040132
101380 0.407989488919667
101478 0.377873065548467
101794 0.358513598419019
102000.666666667 0.353361718859145
102215.666666667 0.416162954894885
102296 0.366299333835124
102619.333333333 0.435495036481021

102638.333333333 0.395285236880308
102778.333333333 0.362671623419761
102793.666666667 0.327620386196036
102806.666666667 0.397510640694224
102909.666666667 0.383371303387617
102971 0.452283483136859
103353 0.399781510894728
103606.666666667 0.349143666077007
103616.666666667 0.420775473589487
103863.666666667 0.471952101800986
104061.666666667 0.409325536612397
104067.666666667 0.365033758213853
104111.333333333 0.409518110146294
104715 0.463251742468114
104730 0.533610133434397
104743.333333333 0.505340641873547
105123.666666667 0.598225414815081
105256.666666667 0.672542071261035
105343.333333333 0.610995838986279
105364 0.538798477458697
105512 0.592746932193807
105631 0.552478773418999
105878.666666667 0.532333214929308
106077.333333333 0.519508774738249
106367 0.481833373443169
107017.666666667 0.566670350301034
107169 0.506822846879274
107187 0.585661513252238
107459.333333333 0.514776121411736
107570.666666667 0.601163972028515
107797.333333333 0.685468140786444
107800 0.609103914741385
107867 0.685005155861379
108147.333333333 0.773217944895772
108182.666666667 0.712742453938194
108349 0.708593407952683
108356 0.682938522189409
108405.666666667 0.606520818903413
108470.666666667 0.661077658102957
108563 0.691856141072448
108765.666666667 0.662525957719299
108951.666666667 0.716913215346899
108952 0.792798530567679
109388.666666667 0.836660507804377
109543.333333333 0.786300245875621
109913 0.705281441234706
109938.666666667 0.765326202887843
110031.333333333 0.724740075139377
110082 0.830707299904276
110089.666666667 0.897864459281423
110230.666666667 0.875930535509963
110300.666666667 0.792190953548653
110305.666666667 0.882595907128124
110321 0.798577156878292
110550 0.728344169356536
110578.333333333 0.693539932263235
110764.666666667 0.737917287441783
111106 0.835110112133172
111444 0.944626727551792
112253.666666667 0.859213981417644
112413 0.940952649544125
112459 0.896794848100413
112768.333333333 0.967133285422885

112896.666666667 0.921008505838007
113792.333333333 0.835514519087366
113859 0.771370883878797
114128.666666667 0.800496301068729
114261.333333333 0.848292547477617
114321 0.831427496706414
114393 0.793269186637818
114614.333333333 0.722017973317685
115162.666666667 0.665483096738923
115218.333333333 0.758548223423822
115282 0.858205432014289
115348.333333333 0.786451760349102
116057.333333333 0.728051501622102
116073.666666667 0.671017641222478
116115 0.758192847457461
116270.666666667 0.843948031397689
116302.666666667 0.838022299445479
116449.333333333 0.82699392899486
116521 0.823413261142148
116980.666666667 0.925731750631438
116982.666666667 0.846288202687814
117031.666666667 0.94581348979929
117095.666666667 0.903113471883069
117126.666666667 0.815964214683247
117294 0.730602232886897
117394.333333333 0.718881342594387
117520.333333333 0.811409177870082
117640.666666667 0.889390360556729
117703 0.801846409904295
117958.333333333 0.733323003279805
118133.666666667 0.683422789439223
118153.666666667 0.736423294341575
118206.666666667 0.716117855187162
118524.333333333 0.670404109601698
118723.666666667 0.619206224260402
118784.666666667 0.574102103013038
119261.666666667 0.64199214981937
119387 0.599993743683846
119391.666666667 0.686056925392924
119565 0.640641764803715
119676.333333333 0.705430647394724
119798 0.76380291836358
119843 0.725823168091973
119932.333333333 0.686925018479579
120048.333333333 0.746270400403886
120114.666666667 0.706868555536005
120324.666666667 0.62258423583202
120341 0.542404159218076
120465.333333333 0.611243302403834
120625.333333333 0.696161785395879
120881 0.663276773335745
120983.333333333 0.658829842067599
121235 0.731507971979767
121437.666666667 0.644957593378811
121598.333333333 0.562319306128389
121670.666666667 0.552085071790353
121693.666666667 0.501453072574565
122029.666666667 0.566621754801901
122131.666666667 0.646645061150615
122164.666666667 0.737690930785076
122325.666666667 0.849702538918978
122396.333333333 0.963054659041054
122431.666666667 0.890338030534088

122515.666666667 0.797090863135993
122597 0.717348907093546
122609.666666667 0.666523604437763
122761 0.755771425392282
123343.666666667 0.693913853450651
123460 0.77945813509787
123896.333333333 0.878559792785113
123898.333333333 0.952635609522836
123995 0.984091297027934
123998.666666667 0.91916398770604
124719.333333333 0.857803246034558
124960 0.852501748710581
